# ChAdOx1 nCoV-19 Vaccine Side Effects among Healthcare Workers in Trinidad and Tobago

**DOI:** 10.3390/vaccines10030466

**Published:** 2022-03-18

**Authors:** Chavin D. Gopaul, Dale Ventour, Davlin Thomas

**Affiliations:** 1 The North Central Regional Health Authority, Eric Williams Medical Sciences Complex, Uriah Butler Highway, Champs Fleur 259807, Trinidad and Tobago; ceo.ncrha@ncrha.co.tt; 2 Anaesthesia & Intensive Care, Faculty of Medical Sciences, The University of the West Indies, St. Augustine 999183, Trinidad and Tobago; dale.ventour@sta.uwi.edu

**Keywords:** vaccine hesitancy, vaccination campaigns, COVID-19 vaccine, ChAdOx1 nCoV-19, adenovirus vaccine, Oxford–AstraZeneca, vaccine safety

## Abstract

Vaccine hesitancy due to safety concerns is a hindrance to the success of vaccination campaigns. In February 2021, Trinidad and Tobago commenced its National COVID-19 Vaccination Program. Healthcare workers were among the first group to receive the ChAdOx1 nCoV-19 (Oxford–AstraZeneca (Covishield, Serum Institute of India, Pune, India), the first COVID-19 vaccine available nationally. This study examined the safety of this vaccine in terms of the systemic and local adverse events following immunization reported by healthcare worker recipients. A cross-sectional study was conducted via a telephone questionnaire. Data concerning demographics, medical and COVID-19-related anamneses, and local and systemic side effects experienced within the first 48 h after receiving the first and second dose of this vaccine, respectively, were gathered. Among the 687 participants (male = 275; female = 412), prevalence of fever, body pain, chills, nausea, myalgia, headache, malaise, fatigue, and other systemic symptoms declined significantly 48 h after administration of the second dose compared to the first dose. Chi-square test and multiple logistic regression demonstrated the greater likelihood of younger recipients to report systemic symptoms compared to older recipients. Multiple logistic regression indicated that females were more likely to report headache, fatigue, and discomfort, and were less likely to report no symptoms, compared to males, after both doses. On average, recipients reported less local and systemic side effects 48 h after receiving the second dose compared to the first dose. The reported rate of occurrence of side effects was <50% for most adverse events, which is consistent with the manufacturer’s claims that the vaccine is safe. This study adds data on the safety of this vaccine in a population that has not been previously studied. The findings can inform public health policy efforts to lower vaccine hesitancy based on safety concerns surrounding the ChAdOx1 nCoV-19 vaccine across various groups in society, including healthcare workers.

## 1. Introduction

The swift development, trial, approval, and rollout of COVID-19 vaccines represent a tremendous achievement by pharmaceutical firms and healthcare professionals [1]. However, a key concern contributing to vaccine hesitancy among the adult population pertains to the fact that there is limited research evidence on the efficacy and safety of the new COVID-19 vaccines, which are currently being administered [2,3]. Initially, reports indicated that some recipients of ChAdOx1 n-COV-19 vaccines had experienced vaccine-induced thrombotic thrombocytopenia (VITT), which forced countries such as the UK to restrict its rollout among the younger population. Therefore, the main issue appears to be the lack of strong research evidence (data) on the safety of the new vaccines, which have been implemented across the globe [1]. This paper seeks to present recent evidence on side effects after administering the ChAdOx1 n-COV-19 vaccines.

Evidence on the safety of the ChAdOx1 n-COV-19 vaccine indicates that the jab is associated with mild local and systemic side effects [1,4,5]. Furthermore, there is evidence that the recipients of the ChAdOx1 n-COV-19 vaccine experience common systemic and local side effects a few days after administration of the vaccine [1]. According to the WHO (2021), common side effects associated with the ChAdOx1 nCoV-19 vaccine include pain and discomfort at the injection site, fatigue, nausea, chills, and muscle pain. However, the likelihood of the stated side effects occurring differs depending on the individual’s age, sex, previous COVID-19 infections, and the type of vaccine administered.

Studies in the Caribbean have been limited to discussing patterns of presented symptoms for SARS-CoV-2 and predictors of ICU admission. Both papers have alluded to the unique characteristics of patients with COVID-19 in Trinidad and Tobago and the greater need for research especially in this region [6,7]. The twin island of the Republic of Trinidad and Tobago has an estimated population of 1.4 million [8]. The country reported its first case of SARS-CoV-2 on 12 March 2020 [9]. Since then, public health measures such as border closures, social distancing, and mask wearing have been implemented to limit the spread of the virus [10]. On 17 February 2021, Trinidad and Tobago joined the global effort to control the pandemic through vaccination when the Ministry of Health embarked upon the Phase 1 rollout of its National COVID-19 Vaccination Program. Healthcare workers were among the first groups to receive the first doses of the ChAdOx1 nCoV-19 (Oxford–AstraZeneca) vaccine, the first brand of COVID-19 vaccine to be made available in the country [11]. As of 31 May 2021, 1179 persons were fully vaccinated (received all doses in a primary vaccine series) and 94,671 persons had received first doses [12].

The aim of this paper is to examine the safety of the first and second dose of the ChAdOx1 n-COV-19 vaccine in terms of the occurrence of systemic and local side effects among healthcare worker recipients 48 h after administration. The eligible participants were the first set of persons to be vaccinated in Trinidad and Tobago’s national vaccination campaign. There are two (2) specific objectives of this research paper. First, the research paper evaluates and compares the safety of the first- and second-dose ChAdOx1 n-COV-19 vaccines within a period of 48 h after administration. Secondly, the paper also evaluates and compares the side effects of the ChAdOx1 n-COV-19 vaccine by age (among persons younger than 40 years and persons older than 40 years) and sex, within a period of 2 days (48 h) after administration.

## 2. Materials and Methods

### 2.1. Setting and Study Design

This cross-sectional study involved the collection of data from 687 healthcare workers (HCWs) of a tertiary care hospital in Trinidad and Tobago, concerning their post-vaccination experience within the first 48 h after receiving the 1st dose and 48 h after receiving the 2nd dose of the ChAdOx1 nCoV-19 vaccine. The HCWs received their 1st doses from 17 February 2021 to 25 February 2021 and 2nd doses from 3 May 2021 to 10 May 2021. The Liaison Unit attached to the hospital conducted telephone calls to interview each HCW 48 h following their receipt of each dose. Specifically, the Liaison Unit conducted these calls from 17 February 2021 to 27 February 2021, and 5 May 2021 to 12 May 2021, to capture data on the adverse events following immunization with the 1st dose and 2nd dose, respectively. A member of the unit entered the de-identified data into a study-specific database. These anonymous data were then solicited through the North Central Regional Health Authority via the Ethics Committee.

The study protocol was reported following the Strengthening the Reporting of Observational Studies in Epidemiology (STROBE) guidelines for cross-sectional studies [13].

### 2.2. Participants

A judgment sampling method was used to obtain the sample of HCWs for this study. The eligible participants were the first group of persons to be vaccinated in Trinidad and Tobago with all primary doses of the ChAdOx1 nCoV-19, which was the first and only COVID-19 vaccine available in the country at the time. HCWs who only received a 1st dose of the vaccine and not a 2nd dose were excluded from this study. A total of 779 HCWs of the hospital received all primary doses of the ChAdOx1 nCoV-19 vaccine regime, i.e., two doses, and were thus eligible to participate in this study. However, only 687 of these HCWs were able to be contacted two days after receipt of the 1st dose as well as two days after receipt of the 2nd dose and agreed to participate in the telephone questionnaire conducted by the Liaison Unit attached to the hospital. The names and contact information of HCWs who received each dose of the vaccine were validated by members of the Liaison Unit who were present at the vaccination stations while the vaccines were being administered to the HCWs. The HCWs’ receipt of the 1st dose of the ChAdOx1 nCoV-19 vaccine was further verified at the outset of the telephone calls to the HCWs, prior to their participation in the telephone survey. HCWs’ receipt of the 2nd dose was further verified in the same manner prior to their participation in the telephone survey. Participation in the survey was voluntary and HCWs received no form of financial remuneration in order to reduce the risk of response bias.

### 2.3. Instrument

The telephone-administered questionnaire consisted of three sections and was developed based on emerging evidence on ChAdOx1 nCoV-19 side effects and was adapted from previous studies [5,14]. The first section included demographic data (age, sex), COVID-19 infection history, and asked for oral verification that the 1st or 2nd dose of the vaccine was received 2 days prior to the telephone call. The second section asked about any side effects experienced within the first forty-eight (48) hours after the 1st dose, while the third asked about any side effects experienced within the first forty-eight (48) hours after the 2nd dose. Section 1 and Section 3 of the questionnaire therefore remained incomplete by each HCW until the telephone call was made 48 h after 2nd dose receipt and responses concerning any side effects experienced after the 2nd dose were filled in. The healthcare workers were asked to orally select the side effects they experienced from a preset list including local (discomfort at injection site) and systemic side effects (fever, body pains, chills, nausea, myalgia, headaches, malaise, fatigue, and other/s). The option for ‘other’ side effects was open-ended and therefore allowed the HCWs to identify any side effect/s they experienced that were not included in the list. This list was compiled from the authors’ judgment based on the relevant literature and adapted from questionnaires used in previous studies investigating COVID-19 vaccine side-effects [5,14]. The telephone questionnaire was pretested among twenty HCWs vaccinated with the first dose of the vaccine to obtain feedback on the clarity of questions and the length of time to complete the questionnaire. The questionnaire was finalized and subsequently conducted among the rest of the HCWs.

### 2.4. Outcome Measures

The outcome measures of this study included the safety of the 1st dose and 2nd dose of the ChAdOx1 nCoV-19 assessed by the occurrence of systemic and local side effects experienced within the first 48 h after receipt of each dose.

### 2.5. Ethics

This study was granted ethical approval by The North Central Regional Health Authority Ethics Committee, Trinidad, and The Ministry of Health of Trinidad and Tobago (3/13/441 Vol. II) Ethics Committee. Participant consent was waived by the ethics committees due to the exclusive use of de-identified data, which were entered into a study-specific database and solicited through the ethics committees. No participant identifiers (name, address, telephone or cell phone number/email/any contact information, ID numbers) were collected or used for the purpose of this study.

### 2.6. Analyses

To examine and compare the safety of the ChAdOx1 n-COV-19 vaccine 48 h after the first and the second dose, descriptive statistical data analysis was first conducted. Specifically, the descriptive statistical analysis entailed presenting the frequency of occurrence of the systemic and local side effects 48 h after the first and second dose.

All analyses were performed using IBM SPSS V.21 software (IBM Corp., Armonk, NY, USA). Non-parametric inferential statistical analysis using Fisher’s exact test and Chi-square test was also conducted in the SPSS statistical package to establish the extent to which the variation in systemic and local side effects by dose (48 h after first and second dose) as well as by age (≤40 years group and >40 years group) was significant. The significance of the difference in systemic and local side effects by dose and age group was assessed at the 1%, 5%, and 10% significance levels. The ANOVA test was conducted to establish whether the difference in the reported number of local and systemic side effects, 48 h after the first and 48 h after the second dose, was statistically significant.

A logistic regression analysis was also conducted to establish the effect of age and gender on the participants’ likelihood of reporting local and systemic side-effects. Specifically, the logistic regression analysis included gender (male and female) as well as age (≤40-year-olds and >40-year-olds) as categorical variables. Each of the local and systemic symptoms was incorporated as a dependent variable whose outcome was influenced by the two independent variables (i.e., age and gender). Specifically, in both 48 h after the first and second dose, there were 12 logistic regression models representing the 11 local and systemic side-effects and the single no symptoms variable. For each of the estimated logistic regression models, age and gender were included as independent (i.e., explanatory) factor variables. The logistic regression analysis was undertaken to supplement the outcome based on the inferential Chi-square test and the ANOVA statistical tests. The significance of age and gender coefficients in the logistic regression analysis was also assessed at 1%, 5% and 10% significance levels.

## 3. Results

### 3.1. Demographic Characteristics

The study included a total of 779 healthcare workers, although only 687 (88.2%) were able to be contacted as 92 (10.2%) respondents were unreachable. A summary on the demographic characteristics of the population is presented in Table 1 below. The participants’ demographic profile indicates that 275 (40%) of the study population were male respondents while most, 412 (60%), were female respondents. In terms of the age profile, 492 (71.6%) of the study population consisted of younger participants aged less than 40 years, with those aged 21–29 years comprising 227 (33%) of the population. The older participants aged above 40 years consisted of those within 41–49 years (118 (17.2%)), 51–59 years (59 (8.6%)), 61–69 years (14 (2.0%)), and 70 years and above (4 (0.6%)). The summary statistics in terms of previous COVID-19 history indicate that only two (*n* = 2) participants had a prior positive case of COVID-19 infection, while most participants (685 (99.7%)) reported no previous COVID-19 infection.

### 3.2. Prevalence of Vaccine Side Effect by Dose, Age, Sex, and Previous COVID-19 History

Table 2 shows the reported adverse side effects within 48 h after the first and second dose of the ChAdOx1 n-COV-19 vaccine. Table 2 indicates that there were significantly less reported cases of systemic symptoms 48 h after the second dose compared to the systemic side effects 48 h after the first dose. Specifically, as compared to the second dose of the ChAdOx1 n-COV-19 vaccine, the most common side effect after the first dose was fever (386 (56.3%)), followed by body pain (314 (45.8%)). The reported prevalence of fever declined from 386 (56.3%) (48 h after first dose) to 103 (15.0%) (48 h after the second dose). Similarly, the reported prevalence of body pain decreased from 314 (45.8%) (48 h after the first dose) to 99 (14.4%) (48 h after the second dose). However, the lowest variation in side effects by the ChAdOx1 n-COV-19 vaccine dose was noted for nausea, where the reported prevalence declined from 70 (10.2%) (48 h after the first dose) to 33 (4.8%) (48 h after the second dose).

Table 2 also shows that the number of local symptoms (discomfort at site) was higher 48 h after the second dose (199 (29.0%)) compared to the reported cases 48 h after the first dose (176 (25.7%)). Furthermore, the reported cases of ‘no symptoms’ increased significantly from 100 (14.6%) (48 h after the first dose) to 276 (40.2%) (48 h after the second dose).

#### 3.2.1. Analysis of the ChAdOx1 n-COV-19 Vaccine Side Effects in First and Second Doses

**Systemic Side Effects**: The results of the descriptive and non-parametric Fisher’s exact test, which were presented for all age groups and both sexes, depicted in Table 2, show that the decrease in the prevalence of fever was statistically significant across the first and the second dose of the ChAdOx1 n-COV-19 vaccine (*p* < 0.0001). Similarly, the decrease in body pain (31.4%; *p* < 0.0001) after the second dose was statistically significant. A summary of the findings also shows that 239 (34.8%) of the participants reported feeling chills 48 h after receiving the first dose, while 33 (4.8%) of the subjects reported experiencing chills 48 h after administration of the ChAdOx1 n-COV-19 s dose. The decrease in the frequency of chills symptoms (30%; *p* < 0.0001) between the first and the second dose of the ChAdOx1 n-COV-19 vaccine was statistically significant. Similarly, the decrease in nausea symptoms (5.4%; *p* < 0.0001) was statistically significant between the first and the second dose.

The descriptive results also indicate that 100 (14.6%) and 23 (3.4%) of the respondents reported experiencing myalgia symptoms 48 h after administration of the first dose and second dose, respectively. The decreased prevalence of myalgia (11.2%; *p* < 0.0001) was statistically significant. Furthermore, the number of participants who reported feeling headache after the first dose (234 (34.1%)) decreased significantly to 110 (16.0%) 48 h after administration of the second dose of the ChAdOx1 n-COV-19 vaccine. The implication is that the decrease in the reported symptom of headache was statistically significant (18.1%; *p* < 0.0001). Similarly, the number of participants who reported feeling malaise (27 (3.9%)) 48 h after receiving the second dose was significantly lower compared to the number of respondents who reported feeling malaise (105 (15.3%)) 48 h after administration of the first dose. The decline in the reported symptom of malaise (11.4%; *p* < 0.0001) between 48 h after the first dose and the second dose was statistically significant. In terms of fatigue, the number of participants who reported feeling tired 48 h after the first dose (207 (30.2%)) was also significantly higher compared to the number of participants (143 (20.9%)) who experienced fatigue within 48 h after receiving the second dose (*p* < 0.0001). The occurrence of the other reported symptoms was also significantly lower (8.6% decrease) 48 h after receiving the second dose (86 (12.5%)) compared to the first dose (145 (21.1%)) (*p* < 0.0001).

**Local Side Effects**: The outcome of the descriptive and non-parametric Fisher’s exact test presented in Table 2 shows that there was a statistically significant difference in the prevalence of the local side effects 48 h after administration of the first and second dose of the ChAdOx1 n-COV-19 vaccine (*p* < 0.0001). Specifically, the Fisher’s exact test results indicate that while 176 (25.7%) of the participants reported feeling discomfort at the site of injection 48 h after the first dose, the increase in reported symptoms after the second dose (199 (29.0%)) was statistically significant (3.3%; *p* < 0.0001). This means that the number of participants who reported local side effects (i.e., discomfort at site) was also significantly different 48 h after administration of the first and second dose of the ChAdOx1 n-COV-19 vaccine. Lastly, ‘no reported symptoms’ also increased significantly 48 h after the second dose (276 (40.2%)) compared to the reported ‘no symptoms’ 48 h after the first dose (100 (14.6%)) (*p* < 0.0001). The implication is that ‘no symptoms’ increased significantly 48 h after administration of the second dose compared to 48 h after administration of the first dose of the ChAdOx1 n-COV-19 vaccine.

The ANOVA test indicated that, on average, the number of reported side effects declined from 3.03 (SD = 1.91) 48 h after the first dose to 1.65 (SD = 1.11) 48 h after administration of the second dose (*p* < 0.0001). This means that, on average, ChAdOx1 n-COV-19 vaccine recipients had less than two (1.65) reported local and systemic side effects 48 h after the second dose compared to the three (3.03) reported after being given the first dose.

#### 3.2.2. Analysis of ChAdOx1 n-COV-19 Side Effects among Younger and Older Recipients

The results in Table 3 show that the two participants who had prior COVID-19 infections were aged below 40 years. The implication is that none of the older respondents (>40 years) reported a positive COVID-19 infection. However, due to the small number of participants who reported prior COVID-19 infections, the Chi-square test results show that there was no statistically significant difference in the COVID-19 anamneses between the younger participants (≤40 years) and the older participants (>40 years).

The results of the descriptive and Chi-square test presented in Table 3 indicate that there was no statistically significant difference in the reported systemic and local side effects in younger (≤40-year-old group) and older (>40-year-old group) ChAdOx1 n-COV-19 vaccine recipients based on the previous COVID-19 history (ρ = 0.373). However, based on the Chi-square test findings, the difference in the reported fatigue symptoms between the ≤40-year-old group (117 (17.1%)) and the >40-year-old group (26 (3.8%)) was statistically significant (*p* = 0.002). The non-parametric inferential results based on the Chi-square test also indicate that the ≤40-year-old group (152 (22.2%)) reported a significantly higher frequency of occurrence of discomfort at the site of injection compared to the >40-year-old group (47 (6.9%)) (*p* = 0.074) when assessed at the 10% significance level. However, more younger participants, ≤40 years (174 (25.4%)), reported no symptoms compared to the older participants, >40 years (102 (14.9%)) (*p* < 0.0001). Therefore, the conclusion based on the inferential Chi-square test findings is that more younger individuals experienced systemic side effects (fatigue) and local side effects (discomfort) compared to the older individuals.

#### 3.2.3. Effect of Age and Gender on ChAdOx1 n-COV-19 Systemic and Local Side Effects

This subsection of the results presents the outcome of the multiple logistic regression analysis to estimate the effect of age and gender on vaccine recipients’ likelihood of experiencing systemic and local side effects. Table 4, Table 5 and Table 6 (first dose panel) show the multiple logistic regression results to ascertain the effect of age and gender on local and systemic side effects 48 h after the first dose. The results indicate that younger age (≤40 years) has significantly higher odds for the likelihood of vaccine recipients reporting systemic and local side effects 48 h after administration of the first dose. Specifically, younger individuals (≤40 years) were 2.60 times (CI 95%: 1.85–3.66) more likely to report fever and 2.50 times (CI 95%: 1.76–3.57) more likely to complain of body pain 48 h after administration of the first dose compared to older vaccine recipients (>40 years). Similarly, younger vaccine recipients (≤40 years) were 2.03 times (CI 95%: 1.39–2.96) more likely to report chills and 2.05 times (CI 95%: 1.07–3.92) more likely to experience nausea symptoms 48 h after the first dose when assessed against the older vaccine recipients (>40 years). The vaccine recipients who were younger than 40 years were 2.51 times (CI 95%: 1.41–4.47) more likely to report myalgia and 1.63 times (CI 95%: 1.12–2.35) more likely to complain of headache 48 h after receiving the first dose compared to older recipients who were aged above 40 years. The multiple logistic regression results also indicate that younger vaccine recipients (≤40 years) were 1.85 times (CI 95%: 1.25–2.73) more likely to report fatigue and 1.45 times (CI 95%: 0.94–2.23) more likely to experience other symptoms 48 h after administration of the first dose compared to older vaccine recipients (>40 years). In addition, older vaccine recipients aged above 40 years had a higher odds ratio of 2.27 times (CI 95%: 1.47–3.52) for reporting no symptoms 48 h after administration of the first dose compared to the younger vaccine recipients (≤40 years).

The outcome of the multiple logistic regression analysis presented in Table 4, Table 5 and Table 6 (first dose panel) indicates that gender has a significantly positive effect on the likelihood of ChAdOx1 n-COV-19 vaccine recipients reporting systemic and local side effects 48 h after the first dose. Specifically, the multiple logistic regression results show that female vaccine recipients were 1.46 times (CI 95%: 1.06–2.00) more likely to complain of body pain and 1.59 times (CI 95%: 1.14–2.22) more likely to experience chills compared to male vaccine recipients, 48 h after administration of the first dose. In addition, female vaccine recipients had odds ratio (OR) of 2.46 times (CI 95%: 1.37–4.39) for experiencing nausea and were 1.74 times (CI 95%: 1.24–2.42) more likely to complain of headache 48 h after administration of the first dose compared to the male vaccine recipients. The female vaccine recipients were also 1.51 times (CI 95%: 1.07–2.13) more likely to report fatigue symptoms and had a higher odds ratio (OR) of 1.41 times (CI 95%: 0.99–2.01) for experiencing discomfort at the site 48 h after administration of the first dose compared to male vaccine recipients. In terms of gender, male vaccine recipients were 1.61 times (CI 95%: 1.05–2.48) more likely to report no symptoms compared to female vaccine recipients 48 h after administration of the first dose.

Table 4, Table 5 and Table 6 (second dose panel) show the multiple logistic regression results to estimate the effect of age and gender on local and systemic side effects 48 h after administration of the second dose of the ChAdOx1 n-COV-19 vaccine. Firstly, 48 h after receiving the second dose, the multiple logistic regression results show that age has a significant effect on the odds of reporting local and systemic side effects. The specific findings indicate that younger vaccine recipients (≤40 years) were 2.06 times (CI 95%: 1.29–3.28) more likely to report fatigue and had a higher adjusted odds ratio of 1.41 times (CI 95%: 0.97–2.07) for experiencing discomfort at the site 48 h after receiving the second dose of the ChAdOx1 n-COV-19 vaccine compared to older vaccine recipients (>40 years). In addition, the multiple logistic regression results indicate that older vaccine recipients aged 40 years and above had higher odds of 2.01 times (CI 95%: 1.44–2.83) of not reporting any symptoms compared to younger vaccine recipients (≤40 years) 48 h after receiving the second dose.

The results of the multiple logistic regression analysis in Table 4, Table 5 and Table 6 (second dose panel) also show that gender has a significantly positive effect on the likelihood of vaccine recipients reporting headache, fatigue, discomfort at site, and no symptoms 48 h after receiving the second dose of the ChAdOx1 n-COV-19 vaccine. Specifically, while female vaccine recipients were 1.69 times (CI 95%: 1.09–2.62) more likely to complain of headache, they also had a greater adjusted odds ratio (AOR) of 2.16 times (CI 95%: 1.43–3.26) for experiencing fatigue symptoms 48 h after receiving the second dose compared to male vaccine recipients. The female vaccine recipients were also 1.52 times (CI 95%: 1.07–2.14) more likely to report discomfort at the site compared to male vaccine recipients, 48 h after administration of the second dose of the ChAdOx1 n-COV-19 vaccine. Finally, male vaccine recipients were 1.68 times (CI 95%: 1.23–2.31) more likely to report no symptoms compared to female vaccine recipients 48 h after administration of the second dose.

## 4. Discussion

The study sought to examine the side effects of the ChAdOx1 n-COV-19 vaccine 48 h after administration of the first and the second dose. The inferential non-parametric test results of the Fisher’s exact test and the Chi-square test analysis reveal that there is a significant difference in the reported systemic symptoms (i.e., fever, body pain, chills, nausea, myalgia, headache, malaise, fatigue, and other systemic symptoms) as well as local symptoms (discomfort at site) 48 h after administration of the first dose and the second dose. The prevalence of reported fever, body pain, chills, nausea, myalgia, headache, malaise, fatigue, and other systemic symptoms declined significantly 48 h after administration of the second dose compared to the reported corresponding systemic symptoms 48 h after administration of the first dose. Similarly, using the ANOVA test, this study also found that the reported number of side effects declined significantly 48 h after the second dose compared to the first dose. Specifically, vaccine recipients reported on average less than two side effects 48 h after the second dose compared to three after the first dose. These results are consistent with previous findings [14] of a similar study that sought to evaluate the safety of the ChAdOx1 n-COV-19 vaccine. According to the study by Menni et al. [14], systemic symptoms, including headache, fatigue, chills, diarrhea, myalgia, nausea, and arthralgia, were 1.6 times more likely to occur after the first dose compared to the second dose, especially among vaccine recipients with prior COVID-19 infections. The study by Bernal et al. [15] also found that recipients of the first dose of the ChAdOx1 n-COV-19 vaccine had a 37% reduced risk of hospital admission due to the occurrence of local and systemic side effects. Similarly, a study examining the side effects of the ChAdOx1 n-COV-19 vaccine in Jordan [16] found that only 32.2% of the vaccine recipients reported mild systemic side effects such as headache, fever, chills, and myalgia. On the other hand, 33.5% of the ChAdOx1 n-COV-19 vaccine recipients experienced moderate local side effects such as pain at the site of injection after administration of the ChAdOx1 n-COV-19 vaccine. However, these findings contrast the outcome of a systematic review study by [17]. The study, which sought to evaluate the safety of the two-dose regimen of ChAdOx1 n-COV-19, found no significant variation in the reported systemic and local side effects after the first and the second dose of the ChAdOx1 n-COV-19 vaccine [17]. In other studies, [18,19], it was also found that there was no significant variation in the reported side effects (headache, fever, myalgia, chills, pain at site, shiver, and nausea) after the first and the second dose of the ChAdOx1 n-COV-19 vaccine. Furthermore, the study conducted by [18] also highlights that the safety of the second dose of the ChAdOx1 n-COV-19 vaccine compared to the first dose varies only by age. Specifically, according to the study by [18], 88% of the 18–55-year-old group reported more systemic side effects (headache, fever, myalgia, chills, and nausea) after receiving the second dose of the ChAdOx1 n-COV-19 vaccine.

The findings of the inferential Chi-square test in this study also reveal that there was substantial variation in the prevalence of the local and systemic side effects among the younger and the older participants. Specifically, the Chi-square test results show that there was a significant difference in the reported prevalence of fatigue, discomfort at site, and no symptoms, 48 h after administration of ChAdOx1 n-COV-19 s dose vaccine among the younger (≤40 years) and the older (>40 years) participants. In addition, the multiple logistic regression results also presented significant evidence that older vaccine recipients have lower odds of reporting fever, body pain, chills, nausea, myalgia, headache, fatigue, and other symptoms 48 h after administration of the first dose.

The multiple logistic regression findings indicated that, 48 h after receiving the second dose, younger vaccine recipients were more likely to experience fever, body pain, fatigue, and discomfort at site compared to older vaccine recipients. Similarly, using multivariate logistic regression analysis to determine the side effects associated with the ChAdOx1 n-COV-19 vaccine in Saudi Arabia, the study by Alhazmi [20] found that only fatigue and fever were significant side effects. Specifically, given that most of the participants in the study by [20] were female, the implication is that gender had a significant effect in determining the prevalence of fever and fatigue side effects after administration of the ChAdOx1 n-COV-19 vaccine. The multiple logistic regression results also depicted that, within 48 h after administration of the first and second dose, the older vaccine (>40) recipients had higher odds of reporting no symptoms compared to younger vaccine recipients. The implication is that younger individuals reported a higher frequency of systemic and local side effects compared to the older participants. The stated insight is also consistent with the previous findings by Abu-Hammad [21], who noted that, among the Jordanian healthcare workers that had received the ChAdOx1 n-COV-19 vaccine, only the age group but not gender was significantly associated with the severity of side effects after the first dose. However, according to [20], neither age nor gender was significantly associated with the severity of the local side effects after administration of the second dose. The insight based on the manufacturer’s trial study also indicated the claim that the ChAdOx1 n-COV-19 vaccine is better tolerated in older recipients compared to younger vaccine recipients with the same immune system [22]. This means that older recipients tend to have less local and systemic side effects compared to younger vaccine recipients.

The stated outcome of the study on the variation in vaccine side effects by age based on Chi-square and multiple logistic regression analysis is also consistent with the study by [18]. According to the stated study, the ChAdOx1 n-COV-19 vaccine seems to be better tolerated in older recipients compared to younger individuals. Specifically, [18] reports that while individuals aged 18–55 years reported 88% prevalence of local side effects, only 61% of those above 70 years complained of experiencing local side effects associated with the ChAdOx1 n-COV-19 vaccine. Madhi et al. [17] also report that younger recipients of the ChAdOx1 n-COV-19 vaccine are more likely to experience systemic and local side effects immediately after vaccination. However, these findings contrast the insight based on the study by Hatmal et al. [16], who did not find any notable differences in the rate of systemic and local side effects among the younger and the older ChAdOx1 n-COV-19 vaccine recipients in Jordan.

The authors of this study found that female respondents are more susceptible to side effects compared to male participants, although the sample composition might have influenced the results. The multiple logistic regression analysis outcome indicated that, 48 h after receiving the first dose, female vaccine recipients had a greater likelihood of complaining of body pain, chills, nausea, headache, fatigue, and discomfort at site compared to male vaccine recipients. The findings also indicated that, 48 h after receiving the second dose, female vaccine recipients also had greater odds of reporting headache, fatigue, and discomfort at site. Similar findings among German healthcare workers by Klugar et al. showed that females were more likely to experience both local and systemic side effects after ChAdOx1 nCoV-19 vaccination than males [23]. A significantly higher prevalence of side effects, including myalgia, fever, and headache, on days one and two after receipt of the first dose of the ChAdOx1 nCoV-19 vaccine was found in females compared to males in a Saudi Arabian sample, with females also experiencing a higher number of side effects than males [24].

The findings have important implications for the public health efforts to raise awareness among the public with respect to vaccine hesitancy. The fact that the reported rate of occurrence of the side effects was mostly less than 50% is consistent with the manufacturer’s claim that the ChAdOx1 n-COV-19 vaccine is safe [22,25]. Furthermore, the insight that the severity and prevalence of the reported side effects decrease 48 h after the second dose compared to 48 h after the first dose suggests that the adverse side effects are mild [26].

## 5. Conclusions

The study concludes and acknowledges that the ChAdOx1 n-COV-19 vaccine is safe, consistent with the manufacturer’s claim. The reported prevalence of the systemic side effects decreases significantly 48 h after administration of the second dose compared to 48 h after administration of the first dose. However, older individuals have lower reported prevalence of systemic and local side effects compared to younger individuals. The implication is that there is a significant difference in the reported systemic and local side effects by age of the vaccine recipients. Specifically, the study found that younger vaccine recipients (≤40 years) reported higher frequencies of fatigue and discomfort at site compared to older vaccine recipients (>40 years). The study also found that the reported prevalence of systemic and local side effects varied significantly by the gender (sex) of the vaccine recipients. On average, female vaccine recipients had a higher reported prevalence of systemic and local side effects 48 h after administration of the first and the second dose compared to male vaccine recipients. Generally, there were more reported systemic side effects compared to local side effects 48 h after administration of the first and the second dose of the ChAdOx1 n-COV-19 vaccine. The findings have important implications for public health policy to lower vaccine hesitancy due to safety concerns.

## Figures and Tables

**Table 1:** Demographic characteristics and COVID-19 history of the study population.

Participant Characteristics	Percent Frequency
Sex:	
Male	275 (40.0%)
Female	412 (60.0%)
Age:	
18–20 Years	2 (0.3%)
21–29 Years	227 (33.0%)
31–39 Years	263 (38.3%)
41–49 Years	118 (17.2%)
51–59 Years	59 (8.6%)
61–69 Years	14 (2.0%)
≥70 Years	4 (0.6%)
Previous COVID-19 History: Yes	2 (0.3%)

**Table 2:** Prevalence of systemic and local adverse side effects by ChAdOx1 n-CoV-19 dose.

Side Effects	ChAdOx1 n-COV-19 First Dose (after 48 h)	ChAdOx1 n-COV-19 Second Dose (after 48 h)	Significance (*p*-Value)
Systemic Side Effects:			
Fever ***	386 (56.3%)	103 (15.0%)	<0.0001
Body Pain ***	314 (45.8%)	99 (14.4%)	<0.0001
Chills ***	239 (34.8%)	33 (4.8%)	<0.0001
Nausea ***	70 (10.2%)	33 (4.8%)	<0.0001
Myalgia ***	100 (14.6%)	23 (3.4%)	<0.0001
Headache ***	234 (34.1%)	110 (16.0%)	<0.0001
Malaise ***	105 (15.3%)	27 (3.9%)	<0.0001
Fatigue ***	207 (30.2%)	143 (20.9%)	<0.0001
Others ***	145 (21.1%)	86 (12.5%)	<0.0001
Local Side Effects:			
Discomfort at Site ***	176 (25.7%)	199 (29.0%)	<0.0001
No Symptoms ***	100 (14.6%)	276 (40.2%)	<0.0001
Number of Side Effects (0–10)	3.03 ± 1.91	1.65 ± 1.11	<0.0001

*** *p* < 0.0001 (*** significant at 0.01% threshold level). Fisher’s exact test was used to compare the difference in the prevalence of systemic and local side effects 48 h after the first and the second dose with a significance level of <0.0001ANOVA test was used to compare the difference between the number of side effects 48 h after the first dose and the second dose with a significance level of <0.0001.

**Table 3:** Prevalence of systemic and local side effects for ChAdOx1 n-COV-19 vaccine among older and younger recipients.

	≤40 Years	>40 Years	Total	Sig. (*p*-Value)
Number of Doses:				
One Dose	491 (71.6%)	195 (28.4%)	686 (100.0%)	<0.0001
Two Doses	491 (71.6%)	195 (28.4%)	686 (100.0%)	
Previous COVID-19 History: Yes	2 (0.3%)	0 (0.0%)	2 (0.3%)	0.373
Systemic Side Effects:				
Fever	80 (11.7%)	23 (3.4%)	103 (15.0%)	0.137
Body Pain	75 (10.4%)	24 (3.5%)	99 (14.4%)	0.318
Chills	24 (3.5%)	9 (1.3%)	33 (4.8%)	0.880
Nausea	22 (3.2%)	11 (1.6%)	33 (4.8%)	0.522
Myalgia	18 (2.6%)	5 (0.7%)	23 (3.4%)	0.470
Headache	81 (11.8%)	29 (4.2%)	110 (16.0%)	0.601
Malaise	20 (2.9%)	7 (1.0%)	27 (3.9%)	0.769
Fatigue ***	117 (17.1%)	26 (3.8%)	143 (20.9%)	0.002
Others	57 (8.3%)	29 (4.2%)	86 (12.5%)	0.244
Local Side Effects:				
Discomfort at Site *	152 (22.2%)	47 (6.9%)	199 (29.0%)	0.074
No Symptoms ***	174 (25.4%)	102 (14.9%)	276 (40.2%)	<0.0001

*** *p* < 0.0001 (*** significant at 0.01% threshold level); * *p* < 0.1 (* significant at 10% threshold level).

**Table 4:** Multiple logistic regression: effect of age and gender on fever, body pain, chills, and nausea side effects.

			Fever	Sig.	Body Pain	Sig.	Chills	Sig.	Nausea	Sig.
			AOR [95% CI]	*p*-Value	AOR [95% CI]	*p*-Value	AOR [95% CI]	*p*-Value	AOR [95% CI]	*p*-Value
First Dose	Age:	≤40 Yrs (vs. >40 Yrs)	2.60 [1.85–3.66]	<0.0001	2.50 [1.76–3.57]	<0.0001	2.03 [1.39–2.96]	<0.0001	2.05 [1.07–3.92]	0.030
	Gender:	Female (vs. Male)	1.04 [0.76–1.43]	0.804	1.46 [1.06–2.00]	0.019	1.59 [1.14–2.22]	0.006	2.46 [1.37–4.39]	0.002
Second Dose	Age:	≤40 Yrs (vs. >40 Yrs)	1.45 [0.89–2.39]	0.140	1.28 [0.78–2.10]	0.321	1.06 [0.48–2.33]	0.881	0.78 [0.37–1.65]	0.516
	Gender:	Female (vs. Male)	1.36 [0.87–2.11]	0.173	1.20 [0.77–1.87]	0.417	1.03 [0.50–2.11]	0.934	1.84 [0.84–4.01]	0.128

**Table 5:** Multiple logistic regression: effect of age and gender on myalgia, headache, malaise, and fatigue side effects.

			Myalgia	Sig.	Headache	Sig.	Malaise	Sig.	Fatigue	Sig.
			AOR [95% CI]	*p*-Value	AOR [95% CI]	*p*-Value	AOR [95% CI]	*p*-Value	AOR [95% CI]	*p*-Value
First Dose	Age:	≤40 Yrs (vs. >40 Yrs)	2.51 [1.41–4.47]	0.002	1.63 [1.12–2.35]	0.010	1.11 [0.70–1.77]	0.663	1.85 [1.25–2.73]	0.002
	Gender:	Female (vs. Male)	1.22 [0.79–1.91]	0.373	1.74 [1.24–2.42]	0.001	0.96 [0.63–1.46]	0.842	1.51 [1.07–2.13]	0.018
Second Dose	Age:	≤40 Yrs (vs. >40 Yrs)	1.45 [0.53–3.95]	0.472	1.13 [0.71–1.79]	0.608	1.14 [0.47–2.74]	0.772	2.06 [1.29–3.28]	0.002
	Gender:	Female (vs. Male)	1.04 [0.44–2.44]	0.927	1.69 [1.09–2.62]	0.019	1.35 [0.60–3.06]	0.467	2.16 [1.43–3.26]	<0.0001

**Table 6:** Multiple logistic regression: effect of age and gender on discomfort at site, other symptoms, and no symptoms regarding side effects.

			Discomfort	Sig.	Other Symptoms	Sig.	No Symptoms	Sig.
			AOR [95% CI]	*p*-Value	AOR [95% CI]	*p*-Value	AOR [95% CI]	*p*-Value
First Dose	Age:	≤40 Yrs (vs. >40 Yrs)	1.04 [0.71–1.52]	0.850	1.45 [0.94–2.23]	0.090	2.27 [1.47–3.52]	<0.0001
	Gender:	Female (vs. Male)	1.41 [0.99–2.01]	0.060	1.16 [0.80–1.70]	0.436	1.61 [1.05–2.48]	0.029
Second Dose	Age:	≤40 Yrs (vs. >40 Yrs)	1.41 [0.97–2.07]	0.076	0.75 [0.47–1.22]	0.246	2.01 [1.44–2.83]	<0.0001
	Gender:	Female (vs. Male)	1.52 [1.07–2.14]	0.019	0.92 [0.58–1.46]	0.724	1.68 [1.23–2.31]	0.001

## Data Availability

The data that support the findings of this study are available from the corresponding author, C.G., upon reasonable request.

## References

[1] Majeed A., Papaluca M., Molokhia M. (2021). Assessing the long-term safety and efficacy of Covid-19 vaccines. J. R. Soc. Med..

[2] Majeed A., Molokhia M. (2020). Vaccinating the UK against Covid-19. BMJ J..

[3] WHO Side-Effects of COVID-19 Vaccines 021. https://www.who.int/news-room/feature-stories/detail/side-effects-of-COVID-19-vaccines/.

[4] Torjesen I. (2021). COVID-19: Norway investigates 23 deaths in frail elderly patients after vaccination. BMJ J..

[5] Riad A., Pokorna A., Attia S., Klugarova J., Koscik M., Klugar M. (2021). Prevalence of Covid-19 vaccine side effects among healthcare workers in Czech Republic. J. Clin. Med..

[6] Gopaul C., Ventour D., Trotman M., Thomas D. (2020). The Epidemiology Characteristics of Positive COVID-19 patients in Trinidad and Tobago. MedRxiv.

[7] Gopaul C., Ventour D., Thomas D. (2020). Laboratory Predictors for COVID-19 ICU Admissions in Trinidad and Tobago. Research Square.

[8] Central Statistical Office (2019). Provisional Mid Year Population Estimate by Age and Sex, 2005–2021. https://cso.gov.tt/subjects/population-and-vital-statistics/population/.

[9] Ministry of Health, Trinidad and Tobago (2020). Statement from the Honourable Terence Deyalsingh, Minister of Health at the Media Conference to Advise of the First Confirmed (Imported) Case of COVID-19 in Trinidad and Tobago. http://www.health.gov.tt/sitepages/default.aspx?id=293.

[10] Government of the Republic of Trinidad and Tobago Ministry of Health COVID-19 Novel Coronavirus. https://health.gov.tt/covid-19/covid-19-guidelines-and-regulations.

[11] Government of the Republic of Trinidad and Tobago Ministry of Health Correction Re: COVID-19 Vaccines Received from Barbados. http://www.news.gov.tt/content/correction-re-covid-19-vaccines-received-barbados#.YeSJ2f7MJEZ.

[12] Government of the Republic of Trinidad and Tobago Ministry of Health Trinidad and Tobago COVID-19 (Novel Coronavirus) Update #741. https://health.gov.tt/covid-19-daily-update-monday-may-31st-2021.

[13] Von Elm E., Altman D.G., Egger M., Pocock S.J., Gotzsche P.C., Vandenbroucke J.P. (2007). The Strengthening the Reporting of Observational Studies in Epidemiology (STROBE) statement: Guidelines for reporting observational studies. Ann. Intern. Med..

[14] Menni C., Klaser K., May A., Polidori L., Capdevila J., Louca P. (2021). Vaccine side-effects and SARS-CoV-2 infection after vaccination in users of the COVID Symptom Study app in the UK: A prospective observational study. Lancet Infect. Dis. J..

[15] Bernal J.L., Andrews N., Gower C., Robertson C., Stowe J., Tessier E. (2021). Effectiveness of the Pfizer-BioNTech and Oxford-AstraZeneca vaccines on covid-19 related symptoms, hospital admissions, and mortality in older adults in England: Test negative case-control study. BMJ J..

[16] Hatmal M.M., Al-Hatamleh M.A., Olaimat A.N., Hatmal M., Alhaj-Qasem D.M., Olaimat T.M., Mohamud R. (2021). Side effects and perceptions following COVID-19 vaccination in Jordan: A randomized, cross-sectional study implementing machine learning for predicting severity of side effects. Vaccines.

[17] Madhi S.A., Baillie V., Cutland C.L., Voysey M., Koen A.L., Fairlie L., Padayachee S.D., Dheda K., Barnabas S.L., Bhorat Q.E. (2021). Safety and efficacy of the ChAdOxI n-Cov-19 (AZDI 222) Covid-19 vaccine against the B.I.35I variant in South Africa. medRxiv.

[18] Ramasamy M.N., Minassian A.M., Ewer K.J., Flaxman A.L., Folegatti P.M., Owens D.R., Voysey M., Aley P.K., Angus B., Babbage G. (2021). Safety and immunogenicity of ChAdOx1 nCoV-19 vaccine administered in a prime-boost regimen in young and old adults (COV002): A single-blind, randomised controlled trial. Lancet J..

[19] Voysey M., Clemens S.A.C., Madhi S.A., Weckx L.Y., Folegatti P.M., Aley P.K., Angus B., Baillie V.L., Barnabas S.L., Bhorat Q.E. (2021). Single-dose administration and the influence of the timing of the booster dose on immunogenicity and efficacy of ChAdOx1 nCoV-19 (AZD1222) vaccine: A pooled analysis of four randomised trials. Lancet J..

[20] Alhazmi A., Alamer E., Daws D., Hakami M., Darraj M., Abdelwahab S., Maghfuri A., Algaissi A. (2021). Evaluation of Side Effects Associated with COVID-19 Vaccines in Saudi Arabia. Vaccines.

[21] Abu-Hammad O., Alduraidi H., Abu-Hammad S., Alnazzawi A., Babkair H., Abu-Hammad A., Nourwali I., Qasem F., Dar-Odeh N. (2021). Side Effects Reported by Jordanian Healthcare Workers Who Received COVID-19 Vaccines. Vaccines.

[22] AstraZeneca (2021). AZD1222 US Phase III Trial Met Primary Efficacy Endpoint in Preventing COVID-19 at Interim Analysis. https://www.astrazeneca.com/.

[23] Klugar M., Riad A., Mekhemar M., Conrad J., Buchbender M., Howaldt H.P., Attia S. (2021). Side effects of mRNA-based and viral vector-based COVID-19 vaccines among German healthcare workers. Biology.

[24] Alghamdi A., Ibrahim A., Almutairi R., Joseph M., Alghamdi G., Alhamza A. (2021). A cross-sectional survey of side effects after COVID-19 vaccination in Saudi Arabia: Male versus female outcomes. J. Adv. Pharm. Educ. Res..

[25] Walsh E.E., Frenck R.W., Falsey A.R., Kitchin N., Absalon J., Gurtman A., Lockhart S., Neuzil K., Mulligan M.J., Bailey R. (2020). Safety and immunogenicity of two RNA-based COVID-19 vaccine candidates. N. Engl. J. Med..

[26] Hazell L., Shakri S.A.W. (2006). Under-reporting of adverse drug reactions. A systematic review. Drug Saf..

